# Response of Phenylpropanoid Pathway and the Role of Polyphenols in Plants under Abiotic Stress

**DOI:** 10.3390/molecules24132452

**Published:** 2019-07-04

**Authors:** Anket Sharma, Babar Shahzad, Abdul Rehman, Renu Bhardwaj, Marco Landi, Bingsong Zheng

**Affiliations:** 1 State Key Laboratory of Subtropical Silviculture, Zhejiang A&F University, Hangzhou 311300, China; 2 School of Land and Food, University of Tasmania, Hobart, TAS 7005, Australia; 3 Department of Crop Science and Biotechnology, Dankook University, Chungnam 31116, Korea; 4 Plant Stress Physiology Laboratory, Department of Botanical and Environmental Sciences, Guru Nanak Dev University, Amritsar 143005, India; 5 Department of Agriculture, Food and Environment, University of Pisa, Via del Borghetto, 80-56124 Pisa, Italy

**Keywords:** abiotic stress, anthocyanin, antioxidant, flavonoid, phenolic acid, polyphenol

## Abstract

Phenolic compounds are an important class of plant secondary metabolites which play crucial physiological roles throughout the plant life cycle. Phenolics are produced under optimal and suboptimal conditions in plants and play key roles in developmental processes like cell division, hormonal regulation, photosynthetic activity, nutrient mineralization, and reproduction. Plants exhibit increased synthesis of polyphenols such as phenolic acids and flavonoids under abiotic stress conditions, which help the plant to cope with environmental constraints. Phenylpropanoid biosynthetic pathway is activated under abiotic stress conditions (drought, heavy metal, salinity, high/low temperature, and ultraviolet radiations) resulting in accumulation of various phenolic compounds which, among other roles, have the potential to scavenge harmful reactive oxygen species. Deepening the research focuses on the phenolic responses to abiotic stress is of great interest for the scientific community. In the present article, we discuss the biochemical and molecular mechanisms related to the activation of phenylpropanoid metabolism and we describe phenolic-mediated stress tolerance in plants. An attempt has been made to provide updated and brand-new information about the response of phenolics under a challenging environment.

## 1. Introduction

Plants face a plethora of biotic and abiotic stresses during their entire life which have negative impact on their growth, development, and productivity [1,2,3]. Biotic factors include insect pests, fungi, and weeds whereas abiotic stresses include salinity, drought, heavy metals, pesticides, ultraviolet (UV) radiation, as well as heat and cold stress [3,4,5,6,7,8,9,10,11,12,13,14,15,16,17,18]. The amplitude of these abiotic stresses has increased severely in recent years principally due to anthropogenic activities [7,19]. Plants, being sessile, are persistently exposed to these factors and require a set of effective mechanisms which can be activated under unfavorable circumstances to sustain their life cycle [20]. According to some reports, the projection of these stresses contributes significantly and affects the growth and productivity by reducing crop yield and overall crop production by 70% and 50%, respectively [21,22]. Thus, it is imperative to reduce the crop productivity losses by improving crop performance through various approaches, including application of plant bio-stimulant products as well as stimulation of plant secondary metabolism [11,23,24]. Plants need to endure different abiotic stresses and polyphenols accumulate in response to these stresses helping plants to acclimatize to unfavorable environments [25,26]. Hence, the concentration of phenols in plant tissue is a good indicator to predict the extent of abiotic stress tolerance in plants which varies greatly in different plant species under an array of external factors.

Phenolic compounds influence the plant growth and development, including seed germination, biomass accumulation, and improved plant metabolism [27,28,29,30]. In this regard, we summarized different studies showing a broad spectrum of different effects of abiotic stresses and discussed how endogenous phenol levels can help in mitigating abiotic stress in plants. Moreover, physiological and molecular mechanisms connected to the phenylpropanoid pathway underlying abiotic stress tolerance have extensively discussed. At the end, we explained phenol-mediated stress tolerance and suggestions have been made to further escalate the extent of deep mechanistic studies.

## 2. Biosynthetic Pathway of Polyphenols in Plants

Phenolics are known to be the largest groups of secondary metabolites in plants varying from simpler aromatic rings to more complex ones, such as lignins. These compounds originated from phenylalanine therefore are also called as phenylpropanoids. Polyphenols are characterized by the presence of large multiples of phenol structural units. The number and characteristics of these phenol structures underlie the unique physical, chemical, and biological properties of particular members of each class. Phenols are indeed divided into several groups such as phenolic acids, flavonoids, stilbenes, and lignans with peculiar properties. Plant phenolics are synthesized biogenetically through a shikimate/phenylpropanoid pathway, whereas a mevalonate pathway generates terpenoids. Both these secondary pathways produce a wide array of monomeric and polymeric structures encompassing a comprehensive array of physiological and biochemical roles in plants. The term “secondary metabolites” refers to the metabolites or phytochemicals synthesized through secondary metabolism. During the biosynthesis of phenolic compounds, erythrose 4-phosphate is combined with phosphoenolpyruvate (PEP) to form phenylalanine. Then phenylalanine ammonia lyase (PAL) catalyzes the conversion of phenylalanine to *trans*-cinnamic acid. Several other phenolic compound such as flavonoids, coumarins, hydrolysable tannins, monolignols, lignans, and lignins are formed through this pathway, formally known as the phenylpropanoid pathway (see complete details in [26,31,32,33]).

## 3. Physiological Roles of Phenolics in Plants

Phenolics are widely distributed and are involved in key metabolic and physiological process in plants [34,35]. Phenolics influence different physiological processes related to growth and development in plants including seed germination, cell division, and synthesis of photosynthetic pigments [36]. Phenolic compounds have been exploited for several application including bioremediation, allelochemical, promotion of plant growth, and antioxidants as food additives [37]. In plants, phenolic accumulation is usually a consistent feature of plants under stress, which represents a defensive mechanism to cope with multiple abiotic stresses [31]. Plant phenolics play an important role in several physiological processes to improve the tolerance and adaptability of plants under suboptimal conditions [38,39,40]. In particular, a large number of secondary metabolites having antioxidant properties belong to this group [41] which can ameliorate plant performance under stress conditions.

Plants interact with their living environment through secondary metabolites. Polyphenols are, for example, involved in signal transduction from the root to the shoot and also help in nutrient mobilization. The roots exudates contain phenolic compounds which alter the physiochemical properties of the rhizosphere. Soil microbes transform phenolics into compounds which help in N mineralization and humus formation [42]. Furthermore, phenolics improve nutrient uptake through chelation of metallic ions, enhanced active absorption sites, and soil porosity with accelerated mobilization of elements like calcium (Ca), magnesium (Mg), potassium (K), zinc (Zn), iron (Fe), and manganese (Mn) [43]. Recently, Rehman, et al. [44] found that Zn application and plant growth promoting rhizobacteria (PGPRs) treatment enhanced the contents of phenolics and organic acids (pyruvic acid, tartaric acid, citric acid, malonic acid, malic acid, succinic acid, oxaloacetic acid, oxalic acid, and methyl malonic acid) in root exudates of wheat, which helped in nutrient mobilization of Zn, N, and Ca and their uptake [44,45]. Phenolic compounds also help in N fixation in legumes. Legumes release several secondary metabolites from roots, principally flavonoid compounds (flavanols and iso-flavonoids) which play crucial role in Nod factors synthesis and in the production of infection tube during nodulation, given that they inhibits auxin transport and facilitate cell division [46].

Plant phenolics, as physiological regulator or chemical messenger, inhibit the IAA catabolism (dihydroxy B-ring flavonoids) or limit the IAA synthesis (monohydroxy B-ring flavonoids) [47]. Flavonoids play a key role in the development of functional pollen. For instance, addition of a very small dose of flavonol aglycones kaempferol or quercetin restored the fertility in mature pollen during pollination [48,49]. Some phenolic compounds (*trans*-cinnamic acid, coumarin, *p*-hydroxybenzoic acid, and benzoic acid) might be potentially phytotoxic if accumulated in high quantity and can inhibit germination and seedling growth [50] due to the disruption of cellular enzyme functioning and impairment of cell division. For instance, some phenolic compounds inhibit the prolyl aminopeptidase and phosphatase enzyme involved in seed germination in legumes [51]. Conversely, high phenolic acid contents have been reported to exert positive effects in seed germinating. In a recent study, Chen et al. [52] found a substantial increase in free (1042%), bound (120%), and total phenolic acid content (741%) in canary grass during germination. The spruce bark extract containing polyphenols stimulated the germination rate in *Lycopersicon esculentum* while inhibited root elongation [53]. Phenolics reduced the thickness and increased the seed tegument porosity which help in water imbibition and boost the germination rate [54]. Polyphenolic extracts of spruce bark intensified the photosynthetic activity and biosynthesis of assimilatory pigment (chlorophyll *a* and *b*) in maize and sunflower [55,56]. Phenolics reduced the energy required for ion transfer by modifying the structure of thylakoids and mitochondrial membranes [57]. As antioxidants, phenolic compounds participate in the scavenging of reactive oxygen species (ROS), catalyzing oxygenation reactions through formation of metallic complexes, and inhibiting the activities of oxidizing enzymes [58]. 

In conclusion, polyphenols are produced under optimal and (with higher levels) in suboptimal conditions in plants and play crucial role in the development encompassing signal transduction, cell division, hormonal regulation, photosynthetic activity regulation, germination, and reproduction rate. Plants exhibiting increased synthesis of polyphenols under abiotic stresses usually show a better adaptability to limiting environments. 

## 4. Abiotic Stresses and Their Toxic Effects on Plants 

**Figure 1:**
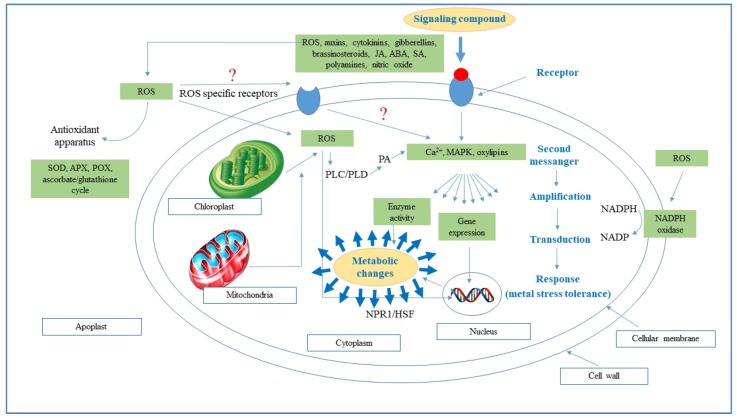
Schematization of signal transmission and transduction in plant cells. Abbreviation: ABA, abscisic acid; APX, ascorbate peroxidase; HSF, redox-sensitive transcription factor; JA, jasmonic acid; MAPK, mitogen-activated protein kinase; NADP, oxidized nicotinamide adenine dinucleotide; NADPH, reduced nicotinamide adenine dinucleotide; NPR1, redox-sensitive transcription factor; OXI1, serine/threonine kinase; PA, phosphatidic acid; PLC/PLD, phospholipases class C and D; POX, peroxidase; ROS, reactive oxygen species; SA, salicylic acid; SOD, superoxide dismutase.

In recent times, producing more food and preventing crop losses to meet the demands of ever-increasing human populations has gained unprecedented importance. Nevertheless, a large proportion of arable land face abiotic stresses (drought, salinity, cold, heat, heavy metal toxicity, UV radiation, etc.) which are expected to increase due to climate change and the incidence of these environmental stresses are further fueled by anthropogenic activities. These abiotic stresses cause alteration in physiological and biochemical processes of plants which results in diminished plant growth and poor yield [59]. These stresses bring rapid changes in cellular redox homeostasis with excessive reactive oxygen species (ROS) generation which eventually damage cell organelles and interfere in ROS-promoted signaling pathways [60]. Contrary to over production of ROS, a physiological redox state hampers normal cell functions and affects the plant immune system, suggesting that a threshold level of ROS is necessary for normal plant functioning (Figure 1; Farooq, et al. [61]). Increased ROS generation under abiotic stresses enhanced itself exponentially the production of ROS [62], which result in peroxidation and destabilization of cellular membranes. Recently Rehman, et al. [63] observed that heat stress and Zn deficiency cause reductions in growth (shoot and root biomass, and root length), and consequently impeded nutrient uptake, enhanced lipid peroxidation and impaired photosynthetic performance. In plants, ROS is produced from 1–2% of total O_2_ consumed in high active cell organelles like chloroplasts, mitochondria, and peroxisomes (Figure 1; [64]). The most common ROS are singlet oxygen (^1^O_2_), superoxide radicle (O_2_^•–^), hydrogen peroxide (H_2_O_2_), and hydroxyl radicle (•OH) [65].

Abiotic stresses disturb the balance between ROS generation and scavenge and accelerate ROS propagation which damages vital macromolecules (nucleic acids, proteins, carbohydrates, and lipids) and eventually leads to cell death. ROS-induced protein damage is caused by oxidation of amino acid residues (e.g., cysteine) for disulphide bond formation, oxidation of arginine, lysine, and threonine residues resulting in irreversible carbonylation in side chains and oxidation of methionine residue to form methionine sulphoxide [66]. ROS production also limits CO_2_ fixation in chloroplasts which are the main site for ROS generation in green plants [67]. ROS reacts with chlorophyll during photosynthesis and forms the chlorophyll triplet state which can rapidly generate (^1^O_2_), thus causing damage to photosynthetic complexes (principally PSII) and perturbing the molecular reaction of the photosynthetic pathway [68]. Apart from the chloroplast, the mitochondria also increase ROS production under abiotic stress which influences plant cellular processes [24]. In mitochondria, ~1–5% of O_2_ consumed leads to H_2_O_2_ formation which is subsequently transformed in •OH during the Fenton reaction [69]. Furthermore, intensive respiratory/photorespiratory metabolism demands high electron input leading to escalated ROS production which results in protein oxidation [61]. Peroxisomes are also major sites for ROS production, particularly H_2_O_2_, and have two- and 50-fold higher concentration of H_2_O_2_ than chloroplasts and mitochondria, respectively [70]. This H_2_O_2_ is involved in stress induced oxidative damage given that it can freely pass lipid membranes. Under the physiological level, different antioxidant defense mechanism detoxify ROS. However, over production of ROS can overwhelm the defense system, resulting in oxidative stress, cell damage, and cell death (Figure 1) [71]. 

## 5. Response and Role of Endogenous Phenolics in Plants against Abiotic Stress

In response to abiotic stresses, biosynthesis of secondary metabolites, including polyphenols, is usually increased in plants. Phenolics confer indeed higher tolerance to plants against various stress conditions like heavy metals, salinity, drought, temperature, pesticides, and UV radiations [33,72,73,74,75,76,77]. Plants growing under stressful environments have the ability to biosynthesize more phenolic compounds in comparison to plants growing under normal conditions [78]. These compounds have antioxidative properties and are capable of scavenging free radicals, resulting in reduction of cell membrane peroxidation [79], hence protecting plant cells from ill effects of oxidative stress. Biosynthesis of phenolics under stressful environments is regulated by the altered activities of various key enzymes of phenolic biosynthetic pathways like PAL and CHS (chalcone synthase). Enhanced performance of enzymes is also accompanied by the up-regulation of the transcript levels of genes encoding key biosynthetic enzymes like *PAL*, *C4H* (cinnamate 4-hydroxylase), *4CL* (4-coumarate: CoA ligase), *CHS*, *CHI* (chalcone isomerase), *F3H* (flavanone3-hydroxylase), *F3′H* (flavonoid 3′-hydroxylase), *F3′5′H* (flavonoid 3′5′-hydroxylase), *DFR* (dihydroflavonol 4-reductase), *FLS* (flavonol synthase), *IFS* (isoflavone synthase), *IFR* (isoflavone reductase), and *UFGT* (UDP flavonoid glycosyltransferase) [74,80,81,82,83,84,85,86]. The responses of phenolic compounds under different abiotic stresses have been discussed in individual sections mentioned below. 

### 5.1. Heavy Metal

Metal stress causes oxidative stress to plants by triggering the generation of harmful ROSs and ultimately cause toxicity and retarded growth [11,87,88]. However, enhanced biosynthesis of phenolics in plants under metal stress helps in protecting plants from oxidative stress [72,89,90]. Flavonoids can enhance the metal chelation process which helps in reducing the levels of harmful hydroxyl radical in plant cells [91,92] and this fits well with the observation that the levels of flavonoids in plants have found to be enhanced by metal excess [90,93]. Under metal toxicity, accumulation of specific flavonoids which are involved in aiding to the plant’s defense mechanism is also enhanced including anthocyanins and flavonols [72,94,95,96]. Accumulation of phenolic compounds is due to the up-regulation of the biosynthesis of phenylpropanoid enzymes including phenylalanine ammonia-lyase, chalcone synthase, shikimate dehydrogenase, cinnamyl alcohol dehydrogenase, and polyphenol oxidase [95,97], which in turn, is dependent on the modulation of transcript levels of genes encoding biosynthetic enzymes under metal stress [72,85]. Flavonoids are also known for their scavenging capability of H_2_O_2_ and are considered to play a crucial role in the phenolic/ascorbate-peroxidase cycle [98,99]. 

Shikimate dehydrogenase (SKDH) and glucose-6-phosphate dehydrogenase (G6PDH) are two important enzymes which catalyze the biological reaction required for the production of important precursors of phenylpropanoid pathways [100]. Another enzyme cinnamyl alcohol dehydrogenase (CADH) catalyzes biochemical reactions which produce precursors required for synthesis of lignin [101]. Heavy metals stimulate phenylpropanoid the biosynthetic pathway in plants by up-regulating the activities of key biosynthetic enzymes like PAL, SKDH, G6PDH, and CADH [101]. Additionally, polyphenol oxidase (PPO) helps during the process of ROS scavenging, and enhancing a plant’s resistance to abiotic stress conditions like heavy metals [100,101,102]. Table 1 summarizes the impact of metal stress on phenolic composition of plants.

### 5.2. Drought 

Phenolic accumulation is very crucial to counteract the negative impacts of drought stress in plants [33]. Transcriptomic and metabolomic studies carried out on *Arabidopsis* plants confirmed that enhanced flavonoid accumulation under drought stress is very helpful to provide resistance [107]. Biosynthesis and accumulation of flavonols were also stimulated in plants under water deficit conditions accompanied by enhanced resistance against drought stress [108,109]. Drought stress also regulated the biosynthetic pathways of phenolic acids and flavonoids, leading to enhanced accumulation of these compounds [82,110,111] which acted as antioxidants and prevented plants from adverse effects of water deficit conditions [112]. For example, contents of flavonoids like kaempferol and quercetin were enhanced in tomato plants accompanied by enhanced drought tolerance [113]. Flavonoid accumulation in cytoplasm can efficiently detoxify harmful H_2_O_2_ molecules generated as a result of drought stress and, at the end oxidation of flavonoids is followed by ascorbic acid mediated re-conversion of flavonoids into primary metabolites [114]. The main reason for this drought-induced accumulation of phenolic compounds is the modulation of phenylpropanoid biosynthetic pathway. Drought regulates many key genes encoding main enzymes of phenylpropanoid pathway, which results in stimulated biosynthesis of phenolic compounds. The impact of drought stress on accumulation of phenolics and related processes has been summarized in Table 2.

### 5.3. Salinity

Salt stress results in generation of ROS like superoxide anions, hydrogen peroxide, and hydroxyl ions [126,127] and require activation of well-orchestrated and finely-tuned plants antioxidant system to contrast ROS propagation [128,129]. Phenolic compounds have powerful antioxidant properties and help in scavenging of harmful ROS in plants under salt stress [130,131,132]. Moreover, in response to salt stress, phenylpropanoid biosynthetic pathway gets stimulated and results in production of various phenolic compounds which have strong antioxidative potential [131,133,134]. 

Some genes like *VvbHLH1* are involved in the enhanced production of flavonoids by regulating the genes of the biosynthetic pathways and confer salt tolerance to plants [135,136]. In tobacco plants, *NtCHS1* plays a crucial role in the biosynthesis of flavonoids under salt stress, where accumulation directly favors the scavenging of ROS [130]. Flavone biosynthesis also was enhanced under saline conditions and in *Glycine max*, it was observed that salinity up-regulates the expression of flavone synthase genes, *GmFNSII-1* and *GmFNSII-2* [137]. Some phenolic acids also accumulate in plants under saline conditions including caffeic acid, caftaric acid, cinnamylmalic acid, gallic acid, ferulic acid, and vanillic acid [131,138,139,140]. Biosynthesis of anthocyanins also was promoted in plants growing under saline conditions [141,142]. A detailed explanation about the effect of salt stress on phenolic composition has been provided in Table 3.

### 5.4. UV Light

Exposure of UV-B radiations to plants causes damage to their protein structure, causes harmful mutations to DNA and generates harmful ROS. To counteract the negative effects of UV-B exposure, endogenous phenolics accumulated in plant cells and protect cell components by making a shield under epidermal layer. They further reduce DNA damage by preventing dimerization of thymine along with reducing photo-damage of important enzymes like NAD/NADP [33,148]. Moreover, flavonoids also act as light screens due to their capability of absorbing both visible (anthocyanins) and UV radiations (anthocyanins and colorless flavonoids), hence protecting plants from these harmful radiations [26,149]. This fact was supported by various researchers who observed enhanced biosynthesis of flavonoids in plants under UV radiations, accompanied by enhanced UV absorption and plant tolerance to these radiations [98,150] and powerful antioxidant capacity [151]. Moreover, it is also well known that plants growing at high altitude accumulate more phenolics like flavonoids than plants of a temperate region. This enhanced flavonoid accumulation under high light/UV exposure is because of stimulated flavonoid biosynthetic pathways and their corresponding gene transcript levels [33,83,152,153]. The key genes which are up-regulated in plants upon UV exposure include: *CHS* (chalcone synthase); *CHI* (chalcone isomerase); *FLS* (flavonol synthase); *DFR* (dihydroflavonol 4-reductase); *FHT* (flavanone 3β-hydroxylase), *FGT* (flavonoid glycosyltransferases); and *PAL* (phenylalanine ammonia lyase) [154,155]. It is also believed that UV light also utilizes jasmonate dependent/independent pathways to stimulate the biosynthesis of phenols in plants [156]. Additionally, abscisic acid (ABA) is also known to modulate the phenolic biosynthetic pathway in presence of UV light [157]. Table 4 provides a brief summary about impact of UV exposure on the endogenous phenolic composition of plants. 

### 5.5. Other Abiotic Factors

Other abiotic factors like temperature, nanoparticles, and pesticides also stimulate the endogenous phenolic biosynthesis in plants and help in providing resistance against phytotoxic effects of these abiotic stresses [74,80,153,168,169,170,171]. Phenolic biosynthetic pathways also get activated in plants growing under pesticide stress conditions. This leads to more accumulation of phenolic compounds in plants, which confer resistance to survive against pesticide toxicity [73,170]. This stimulated phenolic biosynthesis is due to the activation of key biosynthetic enzymes and up-regulation of key genes of phenylpropanoid branch, including *PAL* and *CHS* [74,80]. Increased accumulation of anthocyanins in plant leaves promote by application of insecticides also helps in recovery of plant photosynthetic efficiency [172]. Similarly, under temperature stress (both heat and chilling), plants synthesize more phenolic compounds such as anthocyanins, flavonoids, flavonols, and phenolic acids, which ultimately protect plant cells [75,129,168,169,173]. In *Festuca trachyphylla* plants growing under heat stress, enhancement in the phenolic compounds was noticed including 4-hydroxybenzoic acid, benzoic acid, caffeic acid, coumaric acid, cinnamic acid, gallic acid, homovanillic acid, ferulic acid, salicylic acid, and vanillic acid [76]. The increased accumulation of these phenolic compounds is accompanied by enhanced tolerance of *F. trachyphylla* plants against high temperature [76]. In carrot, phenolics like coumaric acid, caffeic acid, and anthocyanins are suggested to prevent heat induced oxidative damage by enhancing their accumulation [174]. Some phenolics like salicylic acid also act as stimulant for phenol biosynthesis in plants under high temperature stress. This leads to enhanced accumulation of phenolic compounds which further help in detoxification of ROS and providing heat resistance to plants [175]. Under chilling stress, phenolic compounds like suberin or lignin start accumulating in plant cell walls which helps in enhancing resistance against chilling stress [176]. This enhanced thickness of cell wall due to phenolic accumulation is beneficial for prevention of chilling injury and cell collapse under cold stress [33]. Stimulated phenolic biosynthesis under low temperature stress is due to the enhanced expression of *PAL*, *CAD* (cinnamylalcohol dehydrogenase), and *HCT* (hydroxycinnamoyl transferase), and increased phenolic levels play crucial role in protection plants against chilling stress [86]. This fact is further supported by the research carried out on peach under chilling stress by Gao et al. [177]. These researchers suggested that 24-epibrassinolide stimulated biosynthesis of phenolics is involved in reduction of heat generated oxidative stress by helping to scavenge of ROS. Table 5 provides a detailed overview about how different abiotic factors affect phenolic metabolism in plants.

## 6. Conclusions

**Figure 2:**
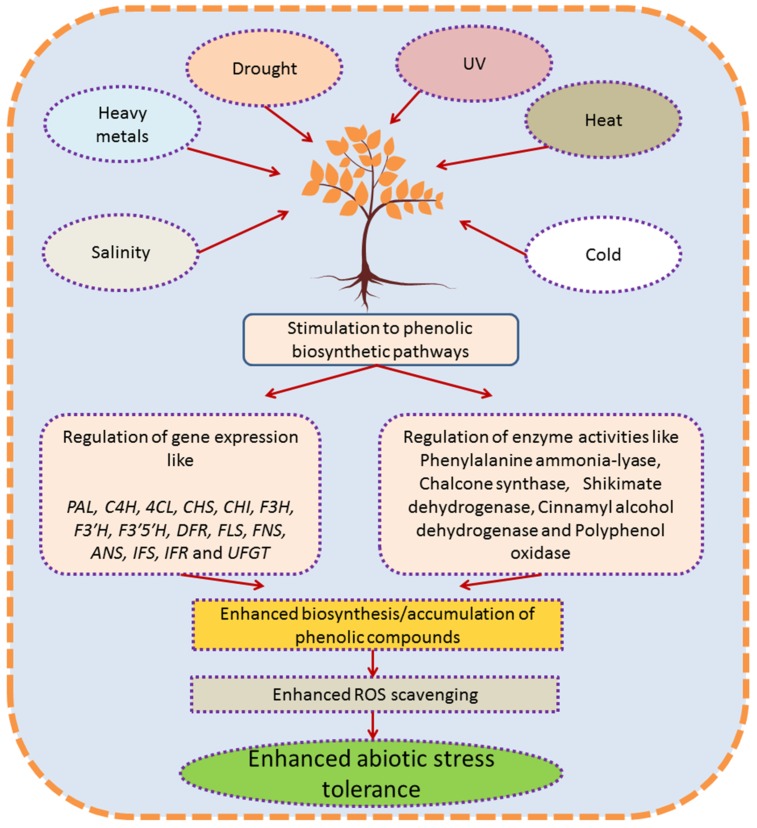
Diagrammatic explanation for response and role of phenolic compounds in plants growing under abiotic stress conditions. ROS (reactive oxygen species); *PAL* (phenylalanine ammonia lyase); *CHS* (chalcone synthase); *CHI* (chalcone isomerase); *C4H* (cinnamate 4-hydroxylase); *4CL* (4-coumarate: CoA ligase); *F3H* (flavanone3-hydroxylase); *F3′H* (flavonoid 3′-hydroxylase); *F3′5′H* (flavonoid 3′5′-hydroxylase); *FLS* (flavonol synthase); *FNS* (flavone synthase) *UFGT* (UDP flavonoid glycosyltransferase); *IFS* (isoflavone synthase); *IFR* (isoflavone reductase); *DFR* (dihydroflavonol 4-reductase); *ANS* (anthocyanidin synthase).

Phenylpropanoid pathway is likely the most studied pathway of secondary metabolism in planta. In plants growing under challenging environments, accumulation of phenolic compounds usually parallels enhanced plant tolerance as summarized in Figure 2. Abiotic stresses also activate the cell signaling process, resulting in transcriptional up-regulation of phenylpropanoid pathway. The increase in plant’s resistance is correlated with the multiple function of polyphenols in plants, principally consisting in their ROS scavenging ability and/or the capacity of some polyphenol classes to protect the plant from excessive light such as UV (flavonoids) and visible light (anthocyanins). In addition, polyphenols might play other key ecological roles under abiotic stress, acting for example as infochemicals for other plants. Aside from the huge body of papers on the matter, further research is needed to deepen, for example, the role of specialized polyphenols as a response to certain abiotic stresses and to describe the intimal mechanisms which shift from primary metabolism to the up-regulation of phenylpropanoid pathway, which is as a cross response to several environmental stressors.

## Figures and Tables

**Table 1:** Summary table describing the impact of heavy metal stress on the endogenous levels of various phenolic compounds in plants.

Plant Species	Heavy Metal	Response of Endogenous Phenolics and Related Parameters	Reference
*Brassica juncea*	Cu	Increase in contents of total phenols, anthocyanins and other phenolic compounds like catechin, caffeic acid, coumaric acid, kaempferol.	[103]
	Cr	Increase in total contents of phenols, flavonoids and anthocyanins, accompanied by enhanced expressions of *PAL* and *CHS*.	[72]
	Cr	Increase in anthocyanins accompanied by up-regulation of *CHS* gene.	[93]
	Cd	Increase in the contents of total flavonoids and anthocyanins.	[90]
	Cd	Increase in total contents of flavonoids and anthocyanins, accompanied by enhanced expressions of *PAL* and *CHS*.	[104]
	Cd	Increase in total contents of phenols, polyphenols, flavonoids and anthocyanins.	[105]
	Pb	Increase in total contents of phenols, flavonoids and anthocyanins, accompanied by enhanced expressions of *PAL* and *CHS*.	[106]
	Pb	Increase in total contents of phenols, polyphenols, flavonoids and anthocyanins.	[89]
*Fagopyrum esculentum*	Al	Increase in total phenolic, flavonoid and anthocyanin contents. Increase in the activity of PAL enzyme.	[77]
*Kandelia obovata*	Cd and Zn	Enhanced levels of total phenolics accompanied by increased activities of phenol metabolic enzymes like shikimate dehydrogenase, cinnamyl alcohol dehydrogenase and polyphenol oxidase.	[97]
*Prosopis farcta*	Pb	Increase in total contents of phenols accompanied by enhanced activity of PAL enzyme. Contents of other phenolic compounds were also increased including ferulic acid, cinnamic acid, caffeic acid, daidzein, vitexin, resveratrol, myricetin, quercetin, kaempferol, naringinine, luteolin and diosmin.	[95]
*Vitis vinifera*	Cu	Enhanced transcript levels of various genes encoding enzymes involved in biosynthesis of phenolics (*PAL*, *C4H*, *CHS*, *F3H*, *DFR*) and down-regulation of *UFGT* and *ANR*.	[85]
*Withania somnifera*	Cd	Increase in total contents of flavonoids and phenolics	[101]
*Zea mays*	Cu, Pb, Cd	Increase in the contents of total phenols and some polyphenols like chlorogenic and vanillic acid.	[96]

PAL (phenylalanine ammonia lyase); CHS (chalcone synthase); CHI (chalcone isomerase); C4H (cinnamate 4-hydroxylase); 4CL (4-coumarate: CoA ligase); F3H (flavanone3-hydroxylase); UFGT (UDP flavonoid glycosyltransferase); IFS (isoflavone synthase); DFR (dihydroflavonol 4-reductase).

**Table 2:** Summary table describing the impact of drought stress on the endogenous levels of various phenolic compounds in plants.

Plant Species	Response of Endogenous Phenolics and Related Parameters	Reference
*Achillea* spp.	Increase in the contents of chlorogenic acid, caffeic acid, rutin, luteolin-*7-O*-glycoside, 1,3-dicaffeoylquinic acid, luteolin, apigenin and kaempferol under 21 days exposure of drought. Enhanced transcript levels of *PAL*, *CHS*, *CHI*, *F3H*, *F3′H*, *F3′5′H* and *FLS*.	[82]
	Increase in contents of total phenols and flavonoids.	[115]
*Brassica napus*	Increase in contents of total phenols, flavonoid and flavonol. Increase in PAL enzyme activity accompanied by enhanced expression of *PAL*.	[110]
*Chrysanthemum morifolium*	Increase in contents of total phenolics, anthocyanins, chlorogenic acid, luteolin, rutin, ferulic acid, apigenin and quercetin. Enhanced expression of *PAL*, *CHI*, and *F3H*, particularly in cultivar Taraneh.	[116]
*Cucumis sativus*	Up-regulation of phenolic metabolites including vanillic acid and 4-hydroxycinnamic acid.	[111]
*Fragaria ananassa*	Enhanced transcript levels of *PAL*, *C4H*, *4CL*, *DFR*, *ANS*, *FLS* and *UFGT*.	[81]
*Lactuca sativa*	Increase in the contents of phenolic compounds such as caftaric acid and rutin.	[117]
*Larrea* spp.	Increase in the contents of polyphenols including flavonoids, proanthocyanidins and flavonols.	[118]
*Lotus japonicus*	Increase in the contents of kaempferol and quercetine. Up-regulation of the expression of *PAL*, *C4H*, *4CL*, *CHS*, *CHI*, *DFR*, *IFS* and *IFR*	[119]
*Nicotiana tabacum*	Increase in PAL enzyme activity and lignin content.	[120]
*Ocimum* spp.	Increase in content of total phenols	[121]
*Thymus vulgaris*	Increase in the contents of total flavonoids and polyphenols.	[122]
*Triticum aestivum*	Increase in content of total phenols	[123]
	Increase in the total contents of phenolics, flavonoids and anthocyanins. Enhanced expression of genes like *CHS*, *CHI*, *F3H*, *FNS*, *FLS*, *DFR* and *ANS*.	[84]
*Vitis vinifera*	Increase in the contents of polyphenols including 4-coumaric acid, caffeic acid, ferulic acid, *cis*-resveratrol-3-O-glucoside, *trans*-resveratrol-3-O-glucoside, catechin, epicatechin, caftaric acid, epicatechin gallate, kaempferol-3-O-glucoside, cyanidin-3-O-glucoside, quercetin-3-O-glucoside and quercetin-3-O glucuronide.	[124]
	Increase in anthocyanin content accompanied by up-regulation of associated biosynthetic genes like *UFGT*, *CHS* and *F3H*.	[125]

PAL (phenylalanine ammonia lyase); CHS (chalcone synthase); CHI (chalcone isomerase); C4H (cinnamate 4-hydroxylase); 4CL (4-coumarate: CoA ligase); F3H (flavanone3-hydroxylase); F3′H (flavonoid 3′-hydroxylase); F3′5′H (flavonoid 3′5′-hydroxylase); FLS (flavonol synthase); FNS (flavone synthase) UFGT (UDP flavonoid glycosyltransferase); IFS (isoflavone synthase); IFR (isoflavone reductase); DFR (dihydroflavonol 4-reductase); ANS (anthocyanidin synthase).

**Table 3:** Summary table describing the impact of salt stress on the endogenous levels of various phenolic compounds in plants.

Plant Species	Response of Endogenous Phenolics and Related Parameters	Reference
*Amaranthus tricolor*	Increase in contents of total phenolics, hydroxybenzoic acids (gallic acid, vanilic acid, syringic acid, *p*-hydroxybenzoic acid, ellagic acid), hydroxycinnamic acids (caffeic acid, chlorogenic acid, *p*-coumaric acid, *m*-coumaric acid, ferulic acid, sinapic acid, *trans*-cinnamic acid) and flavonoids (iso-quercetin, hyperoside, rutin)	[140]
*Asparagus aethiopicus*	Increase in the levels of phenolics like robinin, rutin, apigein, chlorogenic acid and caffeic acid.	[134]
*Carthamus tinctorius*	Increase in contents of total phenols and flavonoids.	[136]
*Chenopodium quinoa*	Increase in total polyphenol and flavonoid contents.	[143]
*Cynara cardunculus*	Increase in contents of phenolic compounds like luteolin-*O*-glucoside, apigenin 6-*c*-glucoside 8-*c*-arabinoside, gallocatechin, leucocyanidin and quercitrin. Decrease in contents of compounds like apigenin, chrysin, genistein, daidzein and ferulic acid	[144]
*Fragaria ananassa*	Enhanced transcript levels of *PAL*, *C4H*, *F3H*, *DFR* and *FLS*.	[81]
*Hordeum vulgare*	Increase of total phenolic contents.	[145]
*Mentha piperita*	Increase of total phenolic contents.	[146]
*Ocimum basilicum*	Increase in the contents of various phenolic compounds like caffeic acis, caftaric acid, cinnamyl malic acid, feruloyl tartaric acid, quercetin-rutinoside and rosmarinic acid.	[139]
*Olea europaea*	Increase in contents of total phenolics, kaempf erol and quercetin. Regulation of transcript levels of *PAL*, *C4H*, *4CL*, *CHS* and *CHI*.	[133]
*Salvia mirzayanii*	Increase of total phenolic contents.	[132]
*Salvia mirzayanii* and *Salvia acrosiphon*	Increase in total phenolic content and PAL activity accompanied by enhanced expression of *PAL*.	[147]
*Solanum lycopersicon*	Increase in total caffeoylquinic acid content	[129]
*Solanum villosum*	Increase in total phenolic, caffeic acid, and quercetin 3-β-D-glucoside contents. Up-regulation of the expression of *PAL* and *FLS*	[138]
*Thymus* spp.	Increase in the contents of various phenolic compounds like caffeic acid, gallic acid, trans-2-hydroxycinnamic acid, cinnamic acid, rosmarinic acid, rutin, syringic acid, vanillic acid, apigenin, quercitrin, naringenin and luteolin.	[131]
*Triticum aestivum*	Increase in contents of total phenols	[123]

PAL (phenylalanine ammonia lyase); CHS (chalcone synthase); CHI (chalcone isomerase); C4H (cinnamate 4-hydroxylase); 4CL (4-coumarate: CoA ligase); F3H (flavanone3-hydroxylase); FLS (flavonol synthase); DFR (dihydroflavonol 4-reductase).

**Table 4:** Summary table describing the impact of UV light exposure on the endogenous levels of various phenolic compounds in plants.

Plant Species	Response of Endogenous Phenolics and Related Parameters	Reference
*Arbutus unedo*	Increase in contents of phenolic compounds like theogallin, avicularin and juglanin.	[158]
*Brassica oleracea*	Increase in contents of gallic acid and sinapic acid.	[159]
*Caryopteris mongolica*	Increase in contents of flavonoids and anthocyanidins, accompanied by PAL and CHI activity.	[160]
*Cuminum cyminum*	Increase in contents of total phenolics and anthocyanins, accompanied by enhanced gene expression of *DAHP* and *PAL*.	[153]
*Fragaria x ananassa*	Increase in contents of kaempferol, ellagic acid and, glucoside derivative of cyaniding, pelargonidin and quercetin. Up-regulation of key genes involved in flavonoid pathway including *CHS*, *CHI*, *FHT*, *DFR*, *FLS* and *FGT*.	[155]
*Kalanchoe pinnata*	Increase in contents of total flavonoids and quercitrin.	[161]
*Lactuca sativa*	Increase in contents of total phenolics, flavonoids and anthocyanins. Contents of phenolic acids were also increased including rosmarinic acid, vanillic acid, *p*-anisic acid, methoxycinnamic acid and chlorogenic acid.	[162]
	Increase in total anthocyanin and phenolic contents. This is accompanied by enhanced activity of PAL enzyme and up-regulation of *PAL* expression.	[163]
*Ribes nigrum*	Increase in contents of flavonols, anthocyanins, hydroxycinnamic and hydroxybenzoic acids.	[164]
*Solanum lycopersicum*	Increase in total phenolic content	[165]
*Triticum aestivum*	After 3 days of UV exposure, increase in contents of total phenolics, ferulic acid, *p*-coumaric acid and vanillic acid, whereas no change in the contents of *p*-hydroxybenzoic acid, syringic acid and sinapic acid. Alterations in the transcript levels of *PAL*, *C4H*, *4CL*, and *COMT*	[83]
*Triticum aestivum*	Increase in contents of free, bound and total phenolics accompanied by enhanced PAL activity.	[166]
*Vigna radiata*	Increase in total flavonoid and phenol content, accompanied by enhanced activities of PAL and CHI enzymes.	[154]
*Vitis vinifera*	Increase in contents of astilbin, quercetin and kaempferol.	[167]
	Increase in contents of phenolic compounds like cyaniding, petunidin, peonidin, malvidin, quercetin, myricetin, kaempferol, procyanidin, gallic acid, protocatechuic acid and vanillic acid.	[157]

CHS (chalcone synthase); CHI (chalcone isomerase); FLS (flavonol synthase); DFR (dihydroflavonol 4-reductase); FHT (flavanone 3β hydroxylase), FGT (flavonoid glycosyltransferases) PAL (phenylalanine ammonia lyase); C4H (cinnamate 4-hydroxylase); 4CL (4-coumarate: CoA ligase); cinnamylalcohol dehydrogenase (CAD); COMT (caffeic acid O-methyltransferase); DAPH (deoxyribonino heptulosinate 7-phosphate synthase).

**Table 5:** Summary table describing the impact of various abiotic factors on the endogenous levels of various phenolic compounds in plants.

Plant Species	Abiotic Factor	Response of Endogenous Phenolics and Related Parameters	Reference
*Brassica juncea*	Insecticide	Increase in total phenol and polyphenol contents.	[73]
	Insecticide	Increase in total phenol, polyphenol and anthocyanin contents accompanied by enhanced expression of *PAL* and *CHS*.	[74]
	Insecticide	Increase in total phenol and anthocyanin contents.	[178]
	Insecticide	Increase in total phenol and anthocyanin contents accompanied by enhanced expression of *PAL* and *CHS*.	[80]
*Dracocephalum kotschyi*	Silicon dioxide NP	Increase in total phenol, total flavonoid, rosmarinic acid and xantomicrol contents, accompanied by up-regulation of the gene expression of *PAL* and *RAS*.	[179]
*Festuca trachyphylla*	Heat	Increase in the contents of phenolic compounds like 4-hydroxybenzoic acid, benzoic acid, caffeic acid, coumaric acid, cinnamic acid, gallic acid, homovanillic acid, ferulic acid, salicylic acid and vanillic acid.	[76]
*Lens culinaris*	Heat	Enhanced levels of total phenolics and flavonoids. Increase in the contents of gallic acid, salicylic acid, chlorogenic acid, ferulic acid and naringenin,	[168]
*Nicotiana tabacum*	Chilling	Alteration in the contents of various metabolites of phenylalanine metabolic pathway. Enhanced expression of *PAL*, *HCT* and *CAD*.	[86]
*Nicotiana langsdorffii*	Heat	Increase in the contents of total polyphenols and individual contents of *p*-coumaric acid, chlorogenic acid, cryptochlorogenic acid, neochlorogenic acid and ferulic acid.	[75]
*Oryza sativa*	Insecticide	Increase in the contents of phenylalanine, p-hydroxybenzoic acid and ferulic acid	[170]
*Prunus persica*	Chilling	Increase in the activities of enzymes like PAL, C4H, 4CL and CHI. Increase in the contents of phenolic compounds like protocatechuic acid, catechin, cholorogenic acid, neocholorogenic acid, quercetin-3- rutinoside, quercetin-3-glucoside, kaempferol-3- rutinoside	[169]
*Solanum lycopersicon*	Heat	Increase in total flavonol content	[129]
	Silver NP	Increase in total phenolic content.	[180]
*Solanum tuberosum*	Zinc NP	Increase in contents of total phenolics and anthocyanins.	[181]
*Vigna angularis*	Heat	Increase in the contents of anthocyanins and flavonoids.	[173]
*Vitis vinifera*	Titanium NP	Increase in contents of total phenolics, caftaric acid, quercetin derivatives and kaempferol derivatives.	[171]
*Withania somnifera*	Copper NP	Increase in contents of total phenolics and flavonoids.	[182]

PAL (phenylalanine ammonia lyase); CHS (chalcone synthase); CHI (chalcone isomerase); C4H (cinnamate 4-hydroxylase); 4CL (4-coumarate: CoA ligase); cinnamylalcohol dehydrogenase (CAD); HCT (hydroxycinnamoyl transferase); COMT (caffeic acid O-methyltransferase); DAPH (deoxyribonino heptulosinate 7-phosphate synthase), RAS (rosmarinic acid synthase); NP (nanoparticles).

## References

[1] Dresselhaus T., Hückelhoven R. (2018). Biotic and Abiotic Stress Responses in Crop Plants. Agronomy.

[2] Lamaoui M., Jemo M., Datla R., Bekkaoui F. (2018). Heat and Drought Stresses in Crops and Approaches for Their Mitigation. Front. Plant Sci..

[3] Mittler R. (2006). Abiotic stress, the field environment and stress combination. Trends Plant Sci..

[4] Kreps J.A., Wu Y., Chang H.-S., Zhu T., Wang X., Harper J.F. (2002). Transcriptome changes for *Arabidopsis* in response to salt, osmotic, and cold stress. Plant Physiol..

[5] Mittler R., Blumwald E. (2010). Genetic engineering for modern agriculture: Challenges and perspectives. Annu. Rev. Plant Biol..

[6] Shao H.-B., Guo Q.-J., Chu L.-Y., Zhao X.-N., Su Z.-L., Hu Y.-C., Cheng J.-F. (2007). Understanding molecular mechanism of higher plant plasticity under abiotic stress. Colloids Surf. B Biointerfaces.

[7] Anjum N.A., Gill S.S., Gill R. (2014). Plant Adaptation to Environmental Change: Significance of Amino Acids and Their Derivatives.

[8] Anjum S.A., Ashraf U., Tanveer M., Khan I., Hussain S., Shahzad B., Zohaib A., Abbas F., Saleem M.F., Ali I. (2017). Drought induced changes in growth, osmolyte accumulation and antioxidant metabolism of three maize hybrids. Front. Plant Sci..

[9] Anjum S.A., Tanveer M., Ashraf U., Hussain S., Shahzad B., Khan I., Wang L. (2016). Effect of progressive drought stress on growth, leaf gas exchange, and antioxidant production in two maize cultivars. Environ. Sci. Pollut. Res..

[10] Anjum S.A., Tanveer M., Hussain S., Bao M., Wang L., Khan I., Ullah E., Tung S.A., Samad R.A., Shahzad B. (2015). Cadmium toxicity in Maize (*Zea mays* L.): Consequences on antioxidative systems, reactive oxygen species and cadmium accumulation. Environ. Sci. Pollut. Res..

[11] Shahzad B., Tanveer M., Che Z., Rehman A., Cheema S.A., Sharma A., Song H., Ur Rehman S., Zhaorong D. (2018). Role of 24-epibrassinolide (EBL) in mediating heavy metal and pesticide induced oxidative stress in plants: A review. Ecotoxicol. Environ. Saf..

[12] Shahzad B., Tanveer M., Rehman A., Cheema S.A., Fahad S., Rehman S., Sharma A. (2018). Nickel; whether toxic or essential for plants and environment-A review. Plant Physiol. Biochem..

[13] Sharma A., Kumar V., Kumar R., Shahzad B., Thukral A.K., Bhardwaj R. (2018). Brassinosteroid-mediated pesticide detoxification in plants: A mini-review. Cogent Food Agric..

[14] Fahad S., Rehman A., Shahzad B., Tanveer M., Saud S., Kamran M., Ihtisham M., Khan S.U., Turan V., Ur Rahman M.H. (2019). Rice Responses and Tolerance to Metal/Metalloid Toxicity. Advances in Rice Research for Abiotic Stress Tolerance.

[15] Soares C., Carvalho M.E., Azevedo R.A., Fidalgo F. (2019). Plants facing oxidative challenges—A little help from the antioxidant networks. Environ. Exp. Bot..

[16] Guo H., Feng X., Hong C., Chen H., Zeng F., Zheng B., Jiang D. (2017). Malate secretion from the root system is an important reason for higher resistance of *Miscanthus sacchariflorus* to cadmium. Physiol. Planta.

[17] Guo H., Chen H., Hong C., Jiang D., Zheng B. (2017). Exogenous malic acid alleviates cadmium toxicity in *Miscanthus sacchariflorus* through enhancing photosynthetic capacity and restraining ROS accumulation. Ecotoxicol. Environ. Saf..

[18] Chen X., Qiu L., Guo H., Wang Y., Yuan H., Yan D., Zheng B. (2017). Spermidine induces physiological and biochemical changes in southern highbush blueberry under drought stress. Braz. J. Bot..

[19] Khan M.I.R., Khan N.A. (2013). Salicylic acid and jasmonates: Approaches in abiotic stress tolerance. J. Plant Biochem. Physiol..

[20] Rao K.M., Raghavendra A., Reddy K.J. (2006). Physiology and Molecular Biology of Stress Tolerance in Plants.

[21] Kaur G., Kumar S., Nayyar H., Upadhyaya H. (2008). Cold stress injury during the pod-filling phase in chickpea (Cicer arietinum L.): Effects on quantitative and qualitative components of seeds. J. Agron. Crop Sci..

[22] Mantri N., Patade V., Penna S., Ford R., Pang E. (2012). Abiotic stress responses in plants: Present and future. Abiotic Stress Responses in Plants.

[23] Shahzad B., Cheema S., Farooq M., Cheema Z., Rehman A., Abbas T. (2018). Growth Stimulating Influence of Foliage Applied *Brassica* Water Extracts on Morphological and Yield Attributes of Bread Wheat under Different Fertilizer Regimes. Planta Daninha.

[24] Sharma P., Jha A.B., Dubey R.S., Pessarakli M. (2012). Reactive Oxygen Species, Oxidative Damage, and Antioxidative Defense Mechanism in Plants under Stressful Conditions. J. Bot..

[25] Pereira A. (2016). Plant abiotic stress challenges from the changing environment. Front. Plant Sci..

[26] Lattanzio V., Ramawat K.G., Mérillon J.-M. (2013). Phenolic Compounds: Introduction. Natural Products: Phytochemistry, Botany and Metabolism of Alkaloids, Phenolics and Terpenes.

[27] Raskin I. (1992). Role of salicylic acid in plants. Ann. Rev. Plant Biol..

[28] Yalpani N., Enyedi A.J., León J., Raskin I. (1994). Ultraviolet light and ozone stimulate accumulation of salicylic acid, pathogenesis-related proteins and virus resistance in tobacco. Planta.

[29] Senaratna T., Touchell D., Bunn E., Dixon K. (2000). Acetyl salicylic acid (Aspirin) and salicylic acid induce multiple stress tolerance in bean and tomato plants. Plant Growth Regul..

[30] Nazar R., Iqbal N., Syeed S., Khan N.A. (2011). Salicylic acid alleviates decreases in photosynthesis under salt stress by enhancing nitrogen and sulfur assimilation and antioxidant metabolism differentially in two mungbean cultivars. J. Plant Physiol..

[31] Cheynier V., Comte G., Davies K.M., Lattanzio V., Martens S. (2013). Plant phenolics: Recent advances on their biosynthesis, genetics, and ecophysiology. Plant Physiol. Biochem..

[32] Saltveit M.E. (2010). Synthesis and metabolism of phenolic compounds. Fruit and Vegetable Phytochemicals Chemistry, Nutritional Value, and Stability.

[33] Naikoo M.I., Dar M.I., Raghib F., Jaleel H., Ahmad B., Raina A., Khan F.A., Naushin F. (2019). Role and Regulation of Plants Phenolics in Abiotic Stress Tolerance: An Overview. Plant Signaling Molecules.

[34] Boudet A.M. (2007). Evolution and current status of research in phenolic compounds. Phytochemistry.

[35] Kumar V., Sharma A., Kohli S.K., Bali S., Sharma M., Kumar R., Bhardwaj R., Thukral A.K. (2019). Differential distribution of polyphenols in plants using multivariate techniques. Biotech. Res. Innov..

[36] Tanase C., Bujor O.-C., Popa V.I., Watson R.R. (2019). Phenolic Natural Compounds and Their Influence on Physiological Processes in Plants. Polyphenols in Plants.

[37] Bujor O.-C., Talmaciu I.A., Volf I., Popa V.I. (2015). Biorefining to recover aromatic compounds with biological properties. TAPPI J..

[38] Andersen C.P. (2003). Source–sink balance and carbon allocation below ground in plants exposed to ozone. New Phytol..

[39] Lattanzio V., Cardinali A., Ruta C., Fortunato I.M., Lattanzio V.M.T., Linsalata V., Cicco N. (2009). Relationship of secondary metabolism to growth in oregano (*Origanum vulgare* L.) shoot cultures under nutritional stress. Environ. Exp. Bot..

[40] Dixon R.A., Paiva N.L. (1995). Stress-induced phenylpropanoid metabolism. Plant Cell.

[41] Oszmanski J. (1995). Polyphenols as antioxidants in food. Przem. Spo..

[42] Halvorson J.J., Gonzalez J.M., Hagerman A.E., Smith J.L. (2009). Sorption of tannin and related phenolic compounds and effects on soluble-N in soil. Soil Biol. Biochem..

[43] Seneviratne G., Jayasinghearachchi H.S. (2003). Mycelial colonization by bradyrhizobia and azorhizobia. J. Biosci..

[44] Rehman A., Farooq M., Naveed M., Nawaz A., Shahzad B. (2018). Seed priming of Zn with endophytic bacteria improves the productivity and grain biofortification of bread wheat. Eur. J. Agron..

[45] Rehman A., Farooq M., Naveed M., Ozturk L., Nawaz A. (2018). *Pseudomonas* aided zinc application improves the productivity and biofortification of bread wheat. Crop Pasture Sci..

[46] Zhang J., Subramanian S., Stacey G., Yu O. (2009). Flavones and flavonols play distinct critical roles during nodulation of *Medicago truncatula* by *Sinorhizobium meliloti*. Plant J..

[47] Mathesius U. (2001). Flavonoids induced in cells undergoing nodule organogenesis in white clover are regulators of auxin breakdown by peroxidase. J. Exp. Bot..

[48] Van Der Meer I.M., Stam M.E., Van Tunen A.J., Mol J.N., Stuitje A.R. (1992). Antisense inhibition of flavonoid biosynthesis in petunia anthers results in male sterility. Plant Cell.

[49] Taylor L.P., Grotewold E. (2005). Flavonoids as developmental regulators. Curr. Opin. Plant Biol..

[50] Baleroni C.R.S., Ferrarese M.L.L., Souza N.E., Ferrarese-Filho O. (2000). Lipid Accumulation during Canola Seed Germination in Response to Cinnamic Acid Derivatives. Biol. Planta.

[51] Shankar S., Girish R., Karthik N., Rajendran R., Mahendran V. (2009). Allelopathic effects of phenolics and terpenoids extracted from Gimelina arborea on germination of Black gram (*Vigna mungo*) and Green gram (*Vigna radiata*). Allelopath. J..

[52] Chen Z., Yu L., Wang X., Gu Z., Beta T. (2016). Changes of phenolic profiles and antioxidant activity in canaryseed (*Phalaris canariensis* L.) during germination. Food Chem..

[53] Balas A., Popa V. Bioactive compounds extracted from *Picea abies* bark. Proceedings of the 10th European Workshop on Lignocellulosics and Pulp.

[54] Tobe K., Zhang L., Qiu G.Y., Shimizu H., Omasa K. (2001). Characteristics of seed germination in five non-halophytic Chinese desert shrub species. J. Arid. Environ..

[55] Tanase C., Boz I., Stingu A., Volf I., Popa V.I. (2014). Physiological and biochemical responses induced by spruce bark aqueous extract and deuterium depleted water with synergistic action in sunflower (*Helianthus annuus* L.) plants. Ind. Crops Prod..

[56] Tanase C., Bara C.I., Popa V.I. (2015). Cytogenetical effect of some polyphenol compounds separated from industrial by-products on. Cell. Chem. Technol..

[57] Moreland D.E., Novitzky W.P. (1987). Effects of Phenolic Acids, Coumarins, and Flavonoids on Isolated Chloroplasts and Mitochondria. Allelochemicals: Role in Agriculture and Forestry.

[58] Amarowicz R., Weidner S. (2009). Biological activity of grapevine phenolic compounds. Grapevine Molecular Physiology & Biotechnology.

[59] Wani S.H., Sah S. (2014). Biotechnology and abiotic stress tolerance in rice. J. Rice Res..

[60] Noctor G., Reichheld J.-P., Foyer C.H. (2018). ROS-related redox regulation and signaling in plants. Semin. Cell Dev. Biol..

[61] Farooq M.A., Niazi A.K., Akhtar J., Farooq M., Souri Z., Karimi N., Rengel Z. (2019). Acquiring control: The evolution of ROS-Induced oxidative stress and redox signaling pathways in plant stress responses. Plant Physiol. Biochem..

[62] Suzuki N., Koussevitzky S., Mittler R., Miller G. (2012). ROS and redox signalling in the response of plants to abiotic stress. Plant Cell Environ..

[63] Rehman A., Farooq M., Asif M., Ozturk L. (2019). Supra-optimal growth temperature exacerbates adverse effects of low Zn supply in wheat. J. Plant Nutr. Soil Sci..

[64] Corpas F.J., Barroso J.B., Palma J.M., Rodriguez-Ruiz M. (2017). Plant peroxisomes: A nitro-oxidative cocktail. Redox Biol..

[65] Gill S.S., Tuteja N. (2010). Reactive oxygen species and antioxidant machinery in abiotic stress tolerance in crop plants. Plant Physiol. Biochem..

[66] Kristensen B.K., Askerlund P., Bykova N.V., Egsgaard H., Moller I.M. (2004). Identification of oxidised proteins in the matrix of rice leaf mitochondria by immunoprecipitation and two-dimensional liquid chromatography-tandem mass spectrometry. Phytochemistry.

[67] Asada K. (2006). Production and Scavenging of Reactive Oxygen Species in Chloroplasts and Their Functions. Plant Physiol..

[68] Buchert F., Forreiter C. (2010). Singlet oxygen inhibits ATPase and proton translocation activity of the thylakoid ATP synthase CF1CFo. FEBS Lett..

[69] Popov V.N., Simonian R.A., Skulachev V.P., Starkov A.A. (1997). Inhibition of the alternative oxidase stimulates H_2_O_2_ production in plant mitochondria. FEBS Lett..

[70] Foyer C.H., Bloom A.J., Queval G., Noctor G. (2009). Photorespiratory metabolism: Genes, mutants, energetics, and redox signaling. Annu. Rev. Plant Biol..

[71] Halliwell B. (2006). Reactive species and antioxidants. Redox biology is a fundamental theme of aerobic life. Plant Physiol..

[72] Handa N., Kohli S.K., Sharma A., Thukral A.K., Bhardwaj R., Abd_Allah E.F., Alqarawi A.A., Ahmad P. (2019). Selenium modulates dynamics of antioxidative defence expression, photosynthetic attributes and secondary metabolites to mitigate chromium toxicity in *Brassica juncea* L. plants. Environ. Exp. Bot..

[73] Sharma A., Kumar V., Thukral A.K., Bhardwaj R. (2016). Epibrassinolide-imidacloprid interaction enhances non-enzymatic antioxidants in *Brassica juncea* L.. Ind. J. Plant Physiol..

[74] Sharma A., Thakur S., Kumar V., Kanwar M.K., Kesavan A.K., Thukral A.K., Bhardwaj R., Alam P., Ahmad P. (2016). Pre-sowing Seed Treatment with 24-Epibrassinolide Ameliorates Pesticide Stress in *Brassica juncea* L. through the Modulation of Stress Markers. Front. Plant Sci..

[75] Ancillotti C., Bogani P., Biricolti S., Calistri E., Checchini L., Ciofi L., Gonnelli C., Del Bubba M. (2015). Changes in polyphenol and sugar concentrations in wild type and genetically modified *Nicotiana langsdorffii* Weinmann in response to water and heat stress. Plant Physiol. Biochem..

[76] Wang J., Yuan B., Huang B. (2019). Differential Heat-Induced Changes in Phenolic Acids Associated with Genotypic Variations in Heat Tolerance for Hard Fescue. Crop Sci..

[77] Smirnov O.E., Kosyan A.M., Kosyk O.I., Taran N.Y. (2015). Response of phenolic metabolism induced by aluminium toxicity in *Fagopyrum esculentum* moench. plants. Ukr. Biochem. J..

[78] Selmar D. (2008). Potential of salt and drought stress to increase pharmaceutical significant secondary compounds in plants. Landbauforschung Volkenrode.

[79] Schroeter H., Boyd C., Spencer J.P., Williams R.J., Cadenas E., Rice-Evans C. (2002). MAPK signaling in neurodegeneration: Influences of flavonoids and of nitric oxide. Neurobiol. Aging.

[80] Sharma A., Yuan H., Kumar V., Ramakrishnan M., Kohli S.K., Kaur R., Thukral A.K., Bhardwaj R., Zheng B. (2019). Castasterone attenuates insecticide induced phytotoxicity in mustard. Ecotoxicol. Environ. Saf..

[81] Perin E.C., Da Silva Messias R., Borowski J.M., Crizel R.L., Schott I.B., Carvalho I.R., Rombaldi C.V., Galli V. (2019). ABA-dependent salt and drought stress improve strawberry fruit quality. Food Chem..

[82] Gharibi S., Sayed Tabatabaei B.E., Saeidi G., Talebi M., Matkowski A. (2019). The effect of drought stress on polyphenolic compounds and expression of flavonoid biosynthesis related genes in *Achillea pachycephala* Rech.f. Phytochemistry.

[83] Chen Z., Ma Y., Yang R., Gu Z., Wang P. (2019). Effects of exogenous Ca2+ on phenolic accumulation and physiological changes in germinated wheat (*Triticum aestivum* L.) under UV-B radiation. Food Chem..

[84] Ma D., Sun D., Wang C., Li Y., Guo T. (2014). Expression of flavonoid biosynthesis genes and accumulation of flavonoid in wheat leaves in response to drought stress. Plant Physiol. Biochem..

[85] Leng X., Jia H., Sun X., Shangguan L., Mu Q., Wang B., Fang J. (2015). Comparative transcriptome analysis of grapevine in response to copper stress. Sci. Rep..

[86] Zhou P., Li Q., Liu G., Xu N., Yang Y., Zeng W., Chen A., Wang S. (2018). Integrated analysis of transcriptomic and metabolomic data reveals critical metabolic pathways involved in polyphenol biosynthesis in *Nicotiana tabacum* under chilling stress. Funct. Plant Biol..

[87] Pandey N., Sharma C.P. (2002). Effect of heavy metals Co^2+^, Ni^2+^ and Cd^2+^ on growth and metabolism of cabbage. Plant Sci..

[88] Villiers F., Ducruix C., Hugouvieux V., Jarno N., Ezan E., Garin J., Junot C., Bourguignon J. (2011). Investigating the plant response to cadmium exposure by proteomic and metabolomic approaches. Proteomics.

[89] Kohli S.K., Handa N., Sharma A., Gautam V., Arora S., Bhardwaj R., Wijaya L., Alyemeni M.N., Ahmad P. (2018). Interaction of 24-epibrassinolide and salicylic acid regulates pigment contents, antioxidative defense responses, and gene expression in *Brassica juncea* L. seedlings under Pb stress. Environ. Sci. Pollut. Res..

[90] Kaur R., Yadav P., Sharma A., Kumar Thukral A., Kumar V., Kaur Kohli S., Bhardwaj R. (2017). Castasterone and citric acid treatment restores photosynthetic attributes in *Brassica juncea* L. under Cd(II) toxicity. Ecotoxicol. Environ. Saf..

[91] Mira L., Fernandez M.T., Santos M., Rocha R., Florencio M.H., Jennings K.R. (2002). Interactions of flavonoids with iron and copper ions: A mechanism for their antioxidant activity. Free Radic. Res..

[92] Williams R.J., Spencer J.P., Rice-Evans C. (2004). Flavonoids: Antioxidants or signalling molecules?. Free. Radic. Biol. Med..

[93] Handa N., Kohli S.K., Sharma A., Thukral A.K., Bhardwaj R., Alyemeni M.N., Wijaya L., Ahmad P. (2018). Selenium ameliorates chromium toxicity through modifications in pigment system, antioxidative capacity, osmotic system, and metal chelators in *Brassica juncea* seedlings. S. Afr. J. Bot..

[94] Trejo-Tapia G., Jimenez-Aparicio A., Rodriguez-Monroy M., De Jesus-Sanchez A., Gutierrez-Lopez G. (2001). Influence of cobalt and other microelements on the production of betalains and the growth of suspension cultures of *Beta vulgaris*. Plant Cell Tiss. Org. Cult..

[95] Zafari S., Sharifi M., Ahmadian Chashmi N., Mur L.A. (2016). Modulation of Pb-induced stress in Prosopis shoots through an interconnected network of signaling molecules, phenolic compounds and amino acids. Plant Physiol. Biochem..

[96] Kısa D., Elmastaş M., Öztürk L., Kayır Ö. (2016). Responses of the phenolic compounds of *Zea mays* under heavy metal stress. Appl. Biol. Chem..

[97] Chen S., Wang Q., Lu H., Li J., Yang D., Liu J., Yan C. (2019). Phenolic metabolism and related heavy metal tolerance mechanism in *Kandelia Obovata* under Cd and Zn stress. Ecotoxicol. Environ. Saf..

[98] Michalak A. (2006). Phenolic compounds and their antioxidant activity in plants growing under heavy metal stress. Pol. J. Environ. Stud..

[99] Keilig K., Ludwig-Mueller J. (2009). Effect of flavonoids on heavy metal tolerance in *Arabidopsis thaliana* seedlings. Bot. Stud..

[100] Kováčik J., Klejdus B., Hedbavny J., Štork F., Bačkor M. (2009). Comparison of cadmium and copper effect on phenolic metabolism, mineral nutrients and stress-related parameters in *Matricaria chamomilla* plants. Plant Soil.

[101] Mishra B., Sangwan N.S. (2019). Amelioration of cadmium stress in *Withania somnifera* by ROS management: Active participation of primary and secondary metabolism. Plant Growth Regul..

[102] Mishra B., Sangwan R.S., Mishra S., Jadaun J.S., Sabir F., Sangwan N.S. (2014). Effect of cadmium stress on inductive enzymatic and nonenzymatic responses of ROS and sugar metabolism in multiple shoot cultures of Ashwagandha (*Withania somnifera* Dunal). Protoplasma.

[103] Poonam R.K., Bhardwaj R., Sirhindi G. (2015). Castasterone regulated polyphenolic metabolism and photosynthetic system in *Brassica juncea* plants under copper stress. J. Pharmacogn. Phytochem..

[104] Kaur P., Bali S., Sharma A., Vig A.P., Bhardwaj R. (2017). Effect of earthworms on growth, photosynthetic efficiency and metal uptake in *Brassica juncea* L. plants grown in cadmium-polluted soils. Environ. Sci. Pollut. Res..

[105] Kaur P., Bali S., Sharma A., Vig A.P., Bhardwaj R. (2018). Role of earthworms in phytoremediation of cadmium (Cd) by modulating the antioxidative potential of *Brassica juncea* L.. Appl. Soil Ecol..

[106] Kohli S.K., Handa N., Sharma A., Kumar V., Kaur P., Bhardwaj R. (2017). Synergistic effect of 24-epibrassinolide and salicylic acid on photosynthetic efficiency and gene expression in *Brassica juncea* L. under Pb stress. Turk. J. Biol..

[107] Nakabayashi R., Yonekura-Sakakibara K., Urano K., Suzuki M., Yamada Y., Nishizawa T., Matsuda F., Kojima M., Sakakibara H., Shinozaki K. (2014). Enhancement of oxidative and drought tolerance in *Arabidopsis* by overaccumulation of antioxidant flavonoids. Plant J..

[108] Kirakosyan A., Seymour E., Kaufman P.B., Warber S., Bolling S., Chang S.C. (2003). Antioxidant capacity of polyphenolic extracts from leaves of *Crataegus laevigata* and *Crataegus monogyna* (Hawthorn) subjected to drought and cold stress. J. Agric. Food Chem..

[109] Ballizany W.L., Hofmann R.W., Jahufer M.Z.Z., Barrett B.A. (2012). Multivariate associations of flavonoid and biomass accumulation in white clover (*Trifolium repens*) under drought. Funct. Plant Biol..

[110] Rezayian M., Niknam V., Ebrahimzadeh H. (2018). Differential responses of phenolic compounds of Brassica napus under drought stress. Iran. J. Plant Physiol..

[111] Li M., Li Y., Zhang W., Li S., Gao Y., Ai X., Zhang D., Liu B., Li Q. (2018). Metabolomics analysis reveals that elevated atmospheric CO_2_ alleviates drought stress in cucumber seedling leaves. Anal. Biochem..

[112] Nichols S.N., Hofmann R.W., Williams W.M. (2015). Physiological drought resistance and accumulation of leaf phenolics in white clover interspecific hybrids. Environ. Exp. Bot..

[113] Sanchez-Rodriguez E., Moreno D.A., Ferreres F., Rubio-Wilhelmi Mdel M., Ruiz J.M. (2011). Differential responses of five cherry tomato varieties to water stress: Changes on phenolic metabolites and related enzymes. Phytochemistry.

[114] Hernandez I., Alegre L., Van Breusegem F., Munne-Bosch S. (2009). How relevant are flavonoids as antioxidants in plants?. Trends Plant Sci..

[115] Gharibi S., Tabatabaei B.E., Saeidi G., Goli S.A. (2016). Effect of Drought Stress on Total Phenolic, Lipid Peroxidation, and Antioxidant Activity of Achillea Species. Appl. Biochem. Biotechnol..

[116] Hodaei M., Rahimmalek M., Arzani A., Talebi M. (2018). The effect of water stress on phytochemical accumulation, bioactive compounds and expression of key genes involved in flavonoid biosynthesis in *Chrysanthemum morifolium* L.. Ind. Crops Prod..

[117] Galieni A., Di Mattia C., De Gregorio M., Speca S., Mastrocola D., Pisante M., Stagnari F. (2015). Effects of nutrient deficiency and abiotic environmental stresses on yield, phenolic compounds and antiradical activity in lettuce (*Lactuca sativa* L.). Sci. Hortic..

[118] Varela M.C., Arslan I., Reginato M.A., Cenzano A.M., Luna M.V. (2016). Phenolic compounds as indicators of drought resistance in shrubs from Patagonian shrublands (Argentina). Plant Physiol. Biochem..

[119] Garcia-Calderon M., Pons-Ferrer T., Mrazova A., Pal’ove-Balang P., Vilkova M., Perez-Delgado C.M., Vega J.M., Eliasova A., Repcak M., Marquez A.J. (2015). Modulation of phenolic metabolism under stress conditions in a *Lotus japonicus* mutant lacking plastidic glutamine synthetase. Front. Plant Sci..

[120] Silva F.L.B., Vieira L.G.E., Ribas A.F., Moro A.L., Neris D.M., Pacheco A.C. (2018). Proline accumulation induces the production of total phenolics in transgenic tobacco plants under water deficit without increasing the G6PDH activity. Theor. Exp. Plant Physiol..

[121] Ghasemi Pirbalouti A., Malekpoor F., Salimi A., Golparvar A. (2017). Exogenous application of chitosan on biochemical and physiological characteristics, phenolic content and antioxidant activity of two species of basil (*Ocimum ciliatum* and *Ocimum basilicum*) under reduced irrigation. Sci. Hortic..

[122] Khalil N., Fekry M., Bishr M., El-Zalabani S., Salama O. (2018). Foliar spraying of salicylic acid induced accumulation of phenolics, increased radical scavenging activity and modified the composition of the essential oil of water stressed *Thymus vulgaris* L.. Plant Physiol. Biochem..

[123] Kaur L., Zhawar V.K. (2015). Phenolic parameters under exogenous ABA, water stress, salt stress in two wheat cultivars varying in drought tolerance. Ind. J. Plant Physiol..

[124] Griesser M., Weingart G., Schoedl-Hummel K., Neumann N., Becker M., Varmuza K., Liebner F., Schuhmacher R., Forneck A. (2015). Severe drought stress is affecting selected primary metabolites, polyphenols, and volatile metabolites in grapevine leaves (*Vitis vinifera* cv. Pinot noir). Plant Physiol. Biochem..

[125] Castellarin S.D., Pfeiffer A., Sivilotti P., Degan M., Peterlunger E., Di Gaspero G. (2007). Transcriptional regulation of anthocyanin biosynthesis in ripening fruits of grapevine under seasonal water deficit. Plant Cell Environ..

[126] Taïbi K., Taïbi F., Ait Abderrahim L., Ennajah A., Belkhodja M., Mulet J.M. (2016). Effect of salt stress on growth, chlorophyll content, lipid peroxidation and antioxidant defence systems in *Phaseolus vulgaris* L.. S. Afr. J. Bot..

[127] Acosta-Motos J.R., Ortuño M.F., Bernal-Vicente A., Diaz-Vivancos P., Sanchez-Blanco M.J., Hernandez J.A. (2017). Plant Responses to Salt Stress: Adaptive Mechanisms. Agronomy.

[128] De Azevedo Neto A.D., Prisco J.T., Enéas-Filho J., Abreu C.E.B.D., Gomes-Filho E. (2006). Effect of salt stress on antioxidative enzymes and lipid peroxidation in leaves and roots of salt-tolerant and salt-sensitive maize genotypes. Environ. Exp. Bot..

[129] Martinez V., Mestre T.C., Rubio F., Girones-Vilaplana A., Moreno D.A., Mittler R., Rivero R.M. (2016). Accumulation of Flavonols over Hydroxycinnamic Acids Favors Oxidative Damage Protection under Abiotic Stress. Front. Plant Sci..

[130] Chen S., Wu F., Li Y., Qian Y., Pan X., Li F., Wang Y., Wu Z., Fu C., Lin H. (2019). NtMYB4 and NtCHS1 Are Critical Factors in the Regulation of Flavonoid Biosynthesis and Are Involved in Salinity Responsiveness. Front. Plant Sci..

[131] Bistgani Z.E., Hashemi M., DaCosta M., Craker L., Maggi F., Morshedloo M.R. (2019). Effect of salinity stress on the physiological characteristics, phenolic compounds and antioxidant activity of *Thymus vulgaris* L. and *Thymus daenensis* Celak. Ind. Crops Prod..

[132] Valifard M., Mohsenzadeh S., Kholdebarin B., Rowshan V. (2014). Effects of salt stress on volatile compounds, total phenolic content and antioxidant activities of *Salvia mirzayanii*. S. Afr. J. Bot..

[133] Rossi L., Borghi M., Francini A., Lin X., Xie D.Y., Sebastiani L. (2016). Salt stress induces differential regulation of the phenylpropanoid pathway in *Olea europaea* cultivars Frantoio (salt-tolerant) and Leccino (salt-sensitive). J. Plant Physiol..

[134] Al-Ghamdi A.A., Elansary H.O. (2018). Synergetic effects of 5-aminolevulinic acid and *Ascophyllum nodosum* seaweed extracts on *Asparagus* phenolics and stress related genes under saline irrigation. Plant Physiol. Biochem..

[135] Golkar P., Taghizadeh M. (2018). In vitro evaluation of phenolic and osmolite compounds, ionic content, and antioxidant activity in safflower (*Carthamus tinctorius* L.) under salinity stress. Plant Cell Tiss. Org. Cult..

[136] Wang F., Zhu H., Chen D., Li Z., Peng R., Yao Q. (2016). A grape bHLH transcription factor gene, VvbHLH1, increases the accumulation of flavonoids and enhances salt and drought tolerance in transgenic *Arabidopsis thaliana*. Plant Cell Tiss. Org. Cult..

[137] Yan J., Wang B., Jiang Y., Cheng L., Wu T. (2014). GmFNSII-controlled soybean flavone metabolism responds to abiotic stresses and regulates plant salt tolerance. Plant Cell Physiol..

[138] Ben-Abdallah S., Zorrig W., Amyot L., Renaud J., Hannoufa A., Lachâal M., Karray-Bouraoui N. (2019). Potential production of polyphenols, carotenoids and glycoalkaloids in *Solanum villosum* Mill. under salt stress. Biologia.

[139] Scagel C.F., Lee J., Mitchell J.N. (2019). Salinity from NaCl changes the nutrient and polyphenolic composition of basil leaves. Ind. Crops Prod..

[140] Sarker U., Oba S. (2018). Augmentation of leaf color parameters, pigments, vitamins, phenolic acids, flavonoids and antioxidant activity in selected *Amaranthus tricolor* under salinity stress. Sci. Rep..

[141] Dkhil B.B., Denden M. (2012). Effect of salt stress on growth, anthocyanins, membrane permeability and chlorophyll fluorescence of Okra (*Abelmoschus esculentus* L.) seedlings. Am. J. Plant Physiol..

[142] Eryılmaz F. (2006). The Relationships between Salt Stress and Anthocyanin Content in Higher Plants. Biotechnol. Biotechnol. Equip..

[143] Aloisi I., Parrotta L., Ruiz K.B., Landi C., Bini L., Cai G., Biondi S., Del Duca S. (2016). New Insight into Quinoa Seed Quality under Salinity: Changes in Proteomic and Amino Acid Profiles, Phenolic Content, and Antioxidant Activity of Protein Extracts. Front. Plant Sci..

[144] Lucini L., Borgognone D., Rouphael Y., Cardarelli M., Bernardi J., Colla G. (2016). Mild Potassium Chloride Stress Alters the Mineral Composition, Hormone Network, and Phenolic Profile in Artichoke Leaves. Front. Plant Sci..

[145] Ma Y., Wang P., Gu Z., Tao Y., Shen C., Zhou Y., Han Y., Yang R. (2019). Ca^2+^ involved in GABA signal transduction for phenolics accumulation in germinated hulless barley under NaCl stress. Food Chem. X.

[146] Çoban Ö., Göktürk Baydar N. (2016). Brassinosteroid effects on some physical and biochemical properties and secondary metabolite accumulation in peppermint (*Mentha piperita* L.) under salt stress. Ind. Crops Prod..

[147] Valifard M., Mohsenzadeh S., Niazi A., Moghadam A. (2015). Phenylalanine ammonia lyase isolation and functional analysis of phenylpropanoid pathway under salinity stress in ‘*Salvia*’ species. Aust. J. Crop Sci..

[148] Daayf F., Lattanzio V. (2009). Recent Advances in Polyphenol Research.

[149] Landi M., Tattini M., Gould K.S. (2015). Multiple functional roles of anthocyanins in plant-environment interactions. Environ. Exp. Bot..

[150] Agati G., Tattini M. (2010). Multiple functional roles of flavonoids in photoprotection. New Phytol..

[151] Agati G., Azzarello E., Pollastri S., Tattini M. (2012). Flavonoids as antioxidants in plants: Location and functional significance. Plant Sci..

[152] Kolb C.A., Kaser M.A., Kopecky J., Zotz G., Riederer M., Pfundel E.E. (2001). Effects of natural intensities of visible and ultraviolet radiation on epidermal ultraviolet screening and photosynthesis in grape leaves. Plant. Physiol..

[153] Ghasemi S., Kumleh H.H., Kordrostami M. (2019). Changes in the expression of some genes involved in the biosynthesis of secondary metabolites in *Cuminum cyminum* L. under UV stress. Protoplasma.

[154] Goyal A., Siddiqui S., Upadhyay N., Soni J. (2014). Effects of ultraviolet irradiation, pulsed electric field, hot water and ethanol vapours treatment on functional properties of mung bean sprouts. J. Food Sci. Technol..

[155] Xu Y., Charles M.T., Luo Z., Mimee B., Veronneau P.Y., Rolland D., Roussel D. (2017). Preharvest Ultraviolet C Irradiation Increased the Level of Polyphenol Accumulation and Flavonoid Pathway Gene Expression in Strawberry Fruit. J. Agric. Food Chem..

[156] Demkura P.V., Abdala G., Baldwin I.T., Ballare C.L. (2010). Jasmonate-dependent and -independent pathways mediate specific effects of solar ultraviolet B radiation on leaf phenolics and antiherbivore defense. Plant Physiol..

[157] Berli F.J., Fanzone M., Piccoli P., Bottini R. (2011). Solar UV-B and ABA are involved in phenol metabolism of *Vitis vinifera* L. increasing biosynthesis of berry skin polyphenols. J. Agric. Food Chem..

[158] Nenadis N., Llorens L., Koufogianni A., Diaz L., Font J., Gonzalez J.A., Verdaguer D. (2015). Interactive effects of UV radiation and reduced precipitation on the seasonal leaf phenolic content/composition and the antioxidant activity of naturally growing *Arbutus unedo* plants. J. Photochem. Photobiol. B.

[159] Moreira-Rodriguez M., Nair V., Benavides J., Cisneros-Zevallos L., Jacobo-Velazquez D.A. (2017). UVA, UVB Light, and Methyl Jasmonate, Alone or Combined, Redirect the Biosynthesis of Glucosinolates, Phenolics, Carotenoids, and Chlorophylls in Broccoli Sprouts. Int. J. Mol. Sci..

[160] Liu M., Cao B., Zhou S., Liu Y. (2012). Responses of the flavonoid pathway to UV-B radiation stress and the correlation with the lipid antioxidant characteristics in the desert plant *Caryopteris mongolica*. Acta Ecol. Sin..

[161] Nascimento L., Leal-Costa M.V., Menezes E.A., Lopes V.R., Muzitano M.F., Costa S.S., Tavares E.S. (2015). Ultraviolet-B radiation effects on phenolic profile and flavonoid content of *Kalanchoe pinnata*. J. Photochem. Photobiol. B.

[162] Sytar O., Zivcak M., Bruckova K., Brestic M., Hemmerich I., Rauh C., Simko I. (2018). Shift in accumulation of flavonoids and phenolic acids in lettuce attributable to changes in ultraviolet radiation and temperature. Sci. Hortic..

[163] Lee M.J., Son J.E., Oh M.M. (2014). Growth and phenolic compounds of *Lactuca sativa* L. grown in a closed-type plant production system with UV-A, -B, or -C lamp. J. Sci. Food Agric..

[164] Huyskens-Keil S., Eichholz I., Kroh L., Rohn S. (2012). UV-B induced changes of phenol composition and antioxidant activity in black currant fruit (*Ribes nigrum* L.). J. App. Bot. Food Qual..

[165] Mariz-Ponte N., Mendes R.J., Sario S., De Oliveira J.F., Melo P., Santos C. (2018). Tomato plants use non-enzymatic antioxidant pathways to cope with moderate UV-A/B irradiation: A contribution to the use of UV-A/B in horticulture. J. Plant Physiol..

[166] Chen Z., Ma Y., Weng Y., Yang R., Gu Z., Wang P. (2019). Effects of UV-B radiation on phenolic accumulation, antioxidant activity and physiological changes in wheat (*Triticum aestivum* L.) seedlings. Food Biosci..

[167] Alonso R., Berli F.J., Fontana A., Piccoli P., Bottini R. (2016). Malbec grape (*Vitis vinifera* L.) responses to the environment: Berry phenolics as influenced by solar UV-B, water deficit and sprayed abscisic acid. Plant Physiol. Biochem..

[168] Swieca M. (2015). Elicitation with abiotic stresses improves pro-health constituents, antioxidant potential and nutritional quality of lentil sprouts. Saudi J. Biol. Sci..

[169] Wang L., Shan T., Xie B., Ling C., Shao S., Jin P., Zheng Y. (2019). Glycine betaine reduces chilling injury in peach fruit by enhancing phenolic and sugar metabolisms. Food Chem..

[170] Mahdavi V., Farimani M.M., Fathi F., Ghassempour A. (2015). A targeted metabolomics approach toward understanding metabolic variations in rice under pesticide stress. Anal. Biochem..

[171] Korosi L., Bouderias S., Csepregi K., Bognar B., Teszlak P., Scarpellini A., Castelli A., Hideg E., Jakab G. (2019). Nanostructured TiO2-induced photocatalytic stress enhances the antioxidant capacity and phenolic content in the leaves of *Vitis vinifera* on a genotype-dependent manner. J. Photochem. Photobiol. B.

[172] Sharma A., Kumar V., Singh R., Thukral A.K., Bhardwaj R. (2016). Effect of seed pre-soaking with 24-epibrassinolide on growth and photosynthetic parameters of *Brassica juncea* L. in imidacloprid soil. Ecotoxicol. Environ. Saf..

[173] Zlotek U., Szymanowska U., Baraniak B., Karas M. (2015). Antioxidant activity of polyphenols of adzuki bean (*Vigna angularis*) germinated in abiotic stress conditions. Acta Sci. Pol. Technol. Aliment..

[174] Commisso M., Toffali K., Strazzer P., Stocchero M., Ceoldo S., Baldan B., Levi M., Guzzo F. (2016). Impact of phenylpropanoid compounds on heat stress tolerance in carrot cell cultures. Front. Plant Sci..

[175] Cingoz G.S., Gurel E. (2016). Effects of salicylic acid on thermotolerance and cardenolide accumulation under high temperature stress in *Digitalis trojana* Ivanina. Plant Physiol. Biochem..

[176] Griffith M., Yaish M.W. (2004). Antifreeze proteins in overwintering plants: A tale of two activities. Trends Plant. Sci..

[177] Gao H., Zhang Z., Lv X., Cheng N., Peng B., Cao W. (2016). Effect of 24-epibrassinolide on chilling injury of peach fruit in relation to phenolic and proline metabolisms. Post. Biol. Technol..

[178] Sharma A., Kumar V., Yuan H., Kanwar M.K., Bhardwaj R., Thukral A.K., Zheng B. (2018). Jasmonic Acid Seed Treatment Stimulates Insecticide Detoxification in *Brassica juncea* L.. Front. Plant Sci..

[179] Nourozi E., Hosseini B., Maleki R., Mandoulakani B.A. (2019). Pharmaceutical important phenolic compounds overproduction and gene expression analysis in *Dracocephalum kotschyi* hairy roots elicited by SiO_2_ nanoparticles. Ind. Crops Prod..

[180] Girilal M., Fayaz A.M., Elumalai L.K., Sathiyaseelan A., Gandhiappan J., Kalaichelvan P.T. (2018). Comparative Stress Physiology Analysis of Biologically and Chemically Synthesized Silver Nanoparticles on *Solanum lycopersicum* L.. Colloid Interface Sci. Commun..

[181] Raigond P., Raigond B., Kaundal B., Singh B., Joshi A., Dutt S. (2017). Effect of zinc nanoparticles on antioxidative system of potato plants. J. Environ. Biol..

[182] Singh O.S., Pant N.C., Laishram M.L., Tewari R.D., Joshi K., Pandey C. (2018). Effect of CuO nanoparticles on polyphenols content and antioxidant activity in Ashwagandha (*Withania somnifera* L. Dunal). J. Pharmacogn. Phytochem..

